# Modulation of caveolins, integrins and plasma membrane repair proteins in anthracycline-induced heart failure in rabbits

**DOI:** 10.1371/journal.pone.0177660

**Published:** 2017-05-12

**Authors:** Yasuhiro Ichikawa, Alice E. Zemljic-Harpf, Zheng Zhang, M. Dan McKirnan, Ana Maria Manso, Robert S. Ross, H. Kirk Hammond, Hemal H. Patel, David M. Roth

**Affiliations:** 1 Veterans Affairs San Diego Healthcare System, San Diego, California, United States of America; 2 Department of Anesthesiology, University of California, San Diego, La Jolla, California, United States of America; 3 Division of Cardiovascular Medicine, Department of Medicine, University of California, San Diego, La Jolla, California, United States of America; Virginia Commonwealth University Medical Center, UNITED STATES

## Abstract

Anthracyclines are chemotherapeutic drugs known to induce heart failure in a dose-dependent manner. Mechanisms involved in anthracycline cardiotoxicity are an area of relevant investigation. Caveolins bind, organize and regulate receptors and signaling molecules within cell membranes. Caveolin-3 (Cav-3), integrins and related membrane repair proteins can function as cardioprotective proteins. Expression of these proteins in anthracycline-induced heart failure has not been evaluated. We tested the hypothesis that daunorubicin alters cardioprotective protein expression in the heart. Rabbits were administered daunorubicin (3 mg/kg, IV) weekly, for three weeks or nine weeks. Nine weeks but not three weeks of daunorubicin resulted in progressive reduced left ventricular function. Cav-3 expression in the heart was unchanged at three weeks of daunorubicin and increased in nine week treated rabbits when compared to control hearts. Electron microscopy showed caveolae in the heart were increased and mitochondrial number and size were decreased after nine weeks of daunorubicin. Activated beta-1 (β1) integrin and the membrane repair protein MG53 were increased after nine weeks of daunorubicin vs. controls with no change at the three week time point. The results suggest a potential pathophysiological role for Cav3, integrins and membrane repair in daunorubicin-induced heart failure.

## Introduction

Cancer remains the second highest cause of mortality in the U.S. [1], behind heart disease. Understanding the wide-range of side effects of cancer therapeutics including cardiotoxicity is essential to optimize therapy and to improve disease and treatment-related survival [2]. Anthracyclines, such as daunorubicin and doxorubicin, are important components of chemotherapy protocols that have been used for over fifty years. Anthracyclines are among the most potent anticancer drugs and are associated with increased patient survival [3]. However, clinical utility is limited by dose-dependent cardiotoxicity that adversely affects 10–30 percent of patients [3]. The morbidity associated with anthracycline-induced cardiotoxicity requires that serial assessment of cardiac function be performed before, during and after treatment. The mechanisms of anthracycline-induced cardiac damage are an important area of investigation [2]. Mechanisms proposed for anthracycline-induced cardiotoxicity include oxidative stress and free radical production, disruption of DNA and RNA synthesis, decreased progenitor cell populations [2,4–6], altered autophagic flux [7], and alterations in multidrug-resistant efflux proteins [8]. Most studies of anthracycline-induced cardiotoxicity have been conducted in rodents [4,7,9]. There is a need for assessment of chemotherapeutics in larger animals to provide clinically relevant data that may elucidate novel mechanisms leading to anthracycline-induced cardiotoxicity.

Caveolae and caveolin proteins are of interest in studies of the pathogenesis of heart failure [10–12] and have not been investigated in anthracycline-induced cardiotoxicity. Caveolae are flask-like invaginations of the plasma membrane that are enriched in cholesterol, sphingolipids, and caveolin proteins (Cav-isoforms 1, 2 and 3) [13]. Cav-3 is the predominant subtype in striated muscle cells and is necessary for caveolae formation in cardiac myocytes [14]. Caveolae, caveolins, and their binding partners including integrins and membrane repair proteins, have been linked to a range of cellular functions including oxidative stress, mitochondrial damage, and alterations in multidrug-resistant protein function. Expression of Cav-3 in the heart reduces oxidative stress and preserves mitochondrial function after ischemia and reperfusion [15]. Drug-sensitive cancer cells display up-regulation of Cav-1 and Cav-2 expression after doxorubicin exposure [16,17]. Our prior work showed that Cav-3 co-localizes with β1D integrin in cardiac myocytes and can modify integrin function [18]. The membrane repair protein mitsugumin-53 (MG53) forms functional complexes with Cav-3 and cavin-1. Cavin-1 is a key molecule in caveolae and membrane repair processes [19] and essential for multi-drug resistance responses in cancer cells [8].

Here we studied anthracycline-cardiotoxicity in rabbits to expand on extensive prior studies in small rodents. We specifically addressed the effects of daunorubicin on caveolae, caveolins, integrins and membrane repair proteins in this clinically relevant model. We hypothesized that Cav-3, integrins and membrane repair proteins would be altered in anthracycline-induced cardiotoxicity. We showed increased myocyte caveolae number and increased expression of Cav-3, activated β1 integrins, and the membrane repair protein MG53 in daunorubicin-induced heart failure. The results suggest that alterations in caveolae and these proteins may be mechanistically involved in daunorubicin -induced cardiotoxicity and therefore warrant further study to identify new avenues to prevent or reduce the limiting side effects of a key group of cancer therapeutics.

## Materials and methods

### Rabbit model of daunorubicin (Dau) cardiotoxicity

Rabbits were handled in compliance with NIH guidelines, the *Guide for the Care and Use of Laboratory Animals* (National Academy of Science). Protocols were approved by the VA San Diego Healthcare System Institutional Animal Care and Use Committee. Male and female New Zealand White rabbits weighing [3.0 ± 0.2 kg (control, n = 8), 3.0 ± 0.1 kg (3 weeks of Dau, n = 6), 3.2 ± 0.1 kg (9 weeks of Dau, n = 8)] were purchased from Western Oregon Rabbit Company (Philomath, OR). Each experimental group included equal numbers of male and female rabbits.

Daunorubicin (Dau) (MP Biomedicals, Santa Ana, CA) was administered as 3 mg/kg, i.v., weekly, for either three weeks (3w Dau) to evaluate early effects of Dau, or for nine weeks (9w Dau) to produce severe cardiotoxicity [20,21]. Echocardiography was performed in the Dau-treated rabbits at pre-injection (time 0) and 6, 7, 8, 9, 10, 11, 12 weeks after the 1^st^ Dau injection (9w Dau) or at pre-injection and 3 weeks after the 1^st^ Dau injection (3w Dau). Cardiac function of all Dau-treated rabbits was serially assessed by echocardiography and rabbits in the nine week Dau group were killed when left ventricular (LV) fractional shortening (FS) was reduced to <20%. Control rabbits were non-treated and sex, age and strain matched. At termination of the study, rabbits were pre-medicated with midazolam (2mg/kg) for 20 minutes and then anesthetized with isoflurane inhalation (5%) and were heparinized with 500 U/kg of heparin. Then rabbits received 10ml of KCL to arrest the hearts in diastole. Hearts were rapidly excised, washed in PBS, and retrogradely perfused with ice-cold saline; weighed and cut into transverse sections for further study.

### Echocardiography

LV systolic function was assessed during the time course of the study using echocardiography via a Philips SONOS 5500 echocardiographic machine with S12 probe (frequency: 5–12 MHz) (Philips, Amsterdam, Netherlands). Rabbits were sedated with midazolam (2 mg/kg, intramuscular) and 1.5% isoflurane administered via inhalation. Guided M-mode imaging was performed at the left ventricular papillary muscle level, and FS was calculated from LV dimensions at end-systole (LVDS) and end-diastole (LVDD) as FS = (LVDD-LVDS)*100/LVDD. LV ejection fraction (EF) was calculated using the Teichholz method [22].

### Western blot and antibodies

Immunoblotting was performed on rabbit heart protein lysates as previously described [23]. Antibodies used were: Cav-1 (3267, Cell Signaling, Danvers, MA), Cav-3 (610421, BD Biosciences, San Jose, CA), DRP1 (sc-32898, Santa Cruz), mitofusin-1 (D6E2S, Cell Signaling), Opa-1 (ab55618, Abcam), talin-1 (Serotec, Raleigh, NC), talin-2 (Serotec), α-tubulin (DHSB, Iowa City, IA), vinculin (V 9131, Sigma, St. Louis, MO), activated β1 integrin (9EG7) (550531, BD Biosciences) MG53 (ab83302, Abcam, Cambridge, MA), and PTRF/cavin-1 (611258, BD Biosciences). Antibodies for β1A andβ1D integrin were kindly provided by Dr. Robert S. Ross [24].

### Electron microscopy

Electron microscopy was performed as previously described [15,25]. Briefly, heart tissues were fixed with 2.5% glutaraldehyde in 0.1 mol/L cacodylate buffer for 2 hours, postfixed in 1% OsO_4_ in 0.1 mol/L cacodylate buffer (1 hour), and embedded as monolayers in LX-112 (Ladd Research, Williston, VTT). Sections were stained in uranyl acetate and lead citrate and were observed with an electron microscope (JEOL 1200 EX-II, JEOL USA, Peabody, Mass; or Philips CM-10, Philips Electronic Instruments, Mahwah, NY). Caveolae were quantified on random images per length of sarcomere.

### Sucrose density membrane fractionation

Whole heart samples were fractionated to isolate caveolar-rich domains using a detergent-free method as previously described [25].

### Histology and immunohistochemistry

Heart tissues were transversely cut and post-fixed by immersion in 4% neutral formaldehyde and embedded in paraffin. Serial sections (6 μm thick) were prepared by conventional histological processing,and stained with hematoxylin and eosin (H&E) and Masson's blue trichrome (for detection of fibrosis). Photomicrographs were imaged using a BZ-X700 microscope (KEYENCE, Osaka, Japan). Fibrosis area and percentage of field were measured by using image-J. Three fields for each rabbit were measured and the mean value of the fields was used to represent the value for each rabbit.

Immunohistochemical analysis was performed as previously described [26]. The specimens were incubated for 5 min in peroxidase-blocking reagent (DAKO Laboratories, Carpinteria, CA) to inactivate endogenous peroxidases. Slides were incubated with primary antibody at 4°C for 24 hours, and then for 30 min in biotinylated mouse anti-goat IgG, and for an additional 30 min in avidin-biotin-horseradish peroxidase complex (both from Vector, Burlingame, CA). The peroxidase reactivity was demonstrated with DAB+, Liquid (DAKO Laboratories).

### Cholesterol assays

Cholesterol in sucrose density fractions was measured with the Amplex Red Cholesterol Assay Kit (Invitrogen) as described by the manufacturer.

### Statistical analysis

Data are presented as mean ± SEM. Statistical analysis was performed between 2 groups using unpaired 2-tailed Student’s t-test or unpaired t-test with Welch’s correction, and among multiple groups using 1-way analysis of variance followed by Tukey’s multiple comparison test. *p*<0.05 was considered significant.

## Results

### Daunorubicin-induced cardiotoxicity

**Fig 1:**
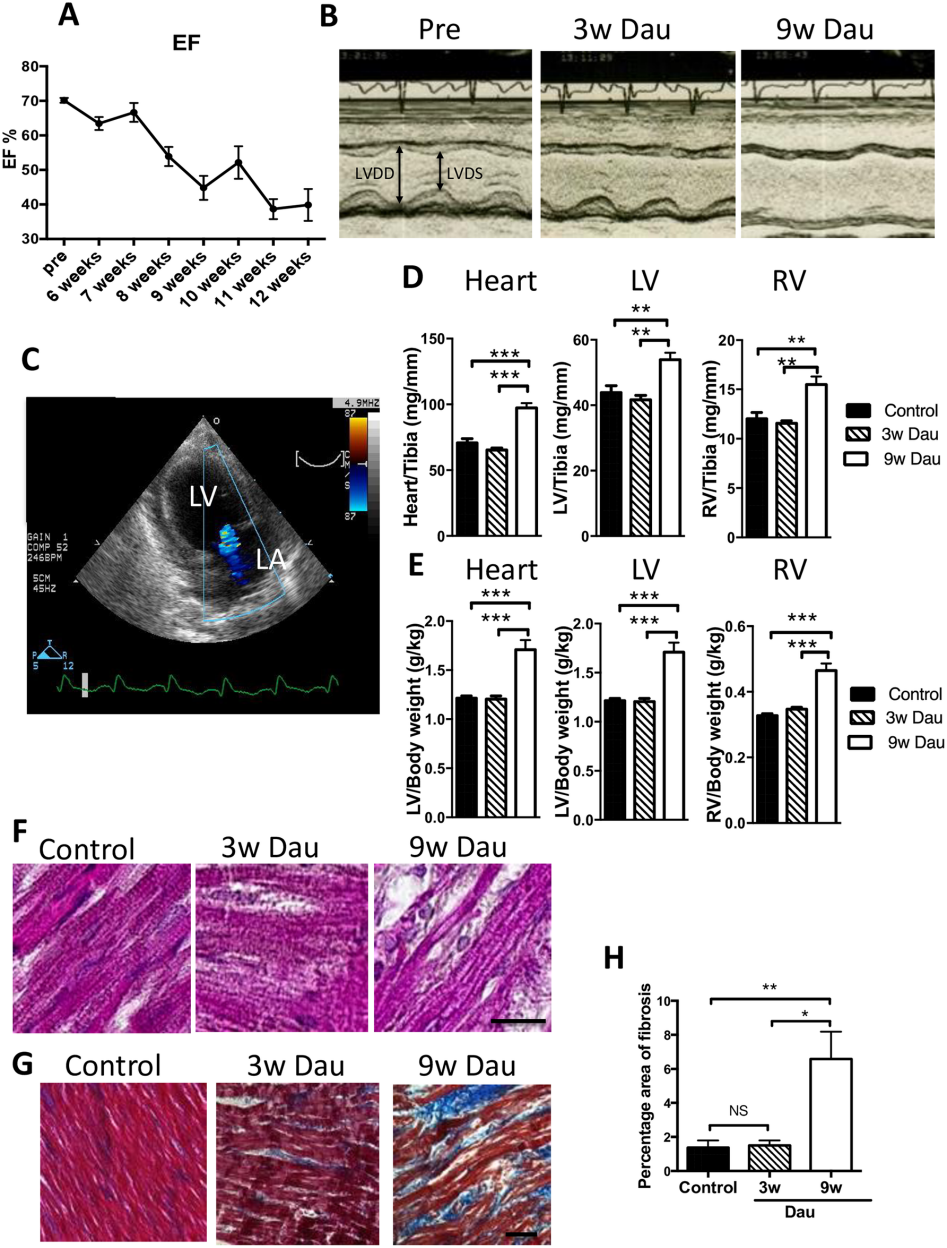
Dau-induced cardiotoxicity in vivo. (A) Ejection fraction (EF) was decreased with 9w Dau treatment. (n = 8) (B) Representative M-mode image of echocardiography before Dau treatment (Pre) and after 3w and 9w of Dau treatment. (C) Mitral regurgitation was found in the 9w Dau-treated rabbits. LA indicates left atrium. (D) Heart to tibia length ratios for Dau-treated and control rabbits (n = 6–8) (E) Heart to body weight (Bw) ratios for Dau-treated and control rabbits (n = 6–8) (F) Hematoxylin and eosin stain of LV from Dau-treated and control hearts (scale bar = 20μm) (G) Masson trichome stain of LV from Dau-treated and control hearts. Blue indicates fibrosis. (scale bar = 50μm) (H) Quantification of (G) (n = 6–8, respectively), *p<0.05, **p<0.01, ***p<0.001, LV, Left Ventricle; RV, Right Ventricle; LVDS and LVDD, LV dimensions in systole and diastole.

In order to develop a model of anthracycline-induced cardiotoxicity in animals larger than rodents, we exposed rabbits to three weeks or nine weeks of daunorubicin (Dau) exposure. Eight of eleven rabbits survived until the end of study in the 9w Dau group and there was no mortality in the 3w Dau group. Nine weeks of Dau treatment resulted in a progressive decline of LV function, as measured by LVEF and LVFS (Fig 1A and Table 1). At the study termination in the nine week Dau group, not only were LVEF and LVFS significantly decreased, but LV chamber dilation was noted when compared to the pre-Dau measurements (Table 1, Fig 1B). Mitral regurgitation (MR) was observed in all rabbits in the 9w Dau group at study end (Fig 1C). In contrast to the 9w Dau group, the 3w Dau group showed no significant changes in LVEF, LVFS, MR, as compared to the pre-treatment evaluation (Table 1).

**Table 1:** Cardiac function in Dau-treated rabbit during echocardiography.

	Pre	3w	Pre	9w
**HR (bpm)**	271 ± 20	232 ± 12	241 ± 9	256 ± 13
**IVSd (mm)**	2.6 ± 0.1	2.7 ± 0.1	2.4 ± 0.1	2.9 ± 0.1*
**LVDd (mm)**	15.5 ± 0.5	16.7 ± 0.6	14.9 ± 0.5	18.9 ± 0.6*
**LVPWd (mm)**	2.8 ± 0.4	2.9 ± 0.3	2.5 ± 0.1	3.2 ± 0.2*
**IVSs (mm)**	3.9 ± 0.2	3.9 ± 0.3	3.6 ± 0.1	3.8 ± 0.2
**LVDs (mm)**	10.6 ± 0.1	11.6 ± 0.6	9.9 ± 0.3	16.1 ± 0.6*&
**LVPWs (mm)**	4.0 ± 0.2	4.2 ± 0.2	3.7 ± 0.3	3.9 ± 0.2
**FS (%)**	31.5 ± 0.5	30.6 ± 0.9	33.3 ± 0.6	14.6 ± 1.0*&
**EF (%)**	67.8 ± 0.7	66.6 ± 0.9	70.2 ± 0.8	37.6 ± 2.2*&

Data are mean ± SEM. HR, heart rate.

IVSd and IVSs, interventricular septal end-diastole and end-systole.

LVDd and LVDs, left ventricular end-diastolic diameter and end-systolic diameter.

LVPWd and LVPWs, LV posterior wall end-diastole and end-systole.

FS, fractional shortening. EF, ejection fraction.

*P < 0.05; Pre vs 9w,

^&^P < 0.05; 3w vs 9w.

At study termination we performed morphometry. Heart weight/body weight and heart weight/tibia length were increased in the 9w Dau-treated rabbits compared to controls. No changes in these morphometric parameters were observed in the 3w Dau group (Fig 1D and 1E). Histology of the hearts of 9w Dau-treated rabbits revealed degenerated cardiomyocytes and vacuolization (Fig 1F). Furthermore, the 9w Dau-treated rabbits had perivascular and interstitial cardiac fibrosis compared with control rabbits and rabbits in the 3w Dau group (Fig 1G and 1H). Thus we established a rabbit model in which the effects of Dau could be evaluated early (3w) when cardiac function was preserved, and later (9w) when heart failure was evident.

### Caveolin expression in the heart after daunorubicin

**Fig 2:**
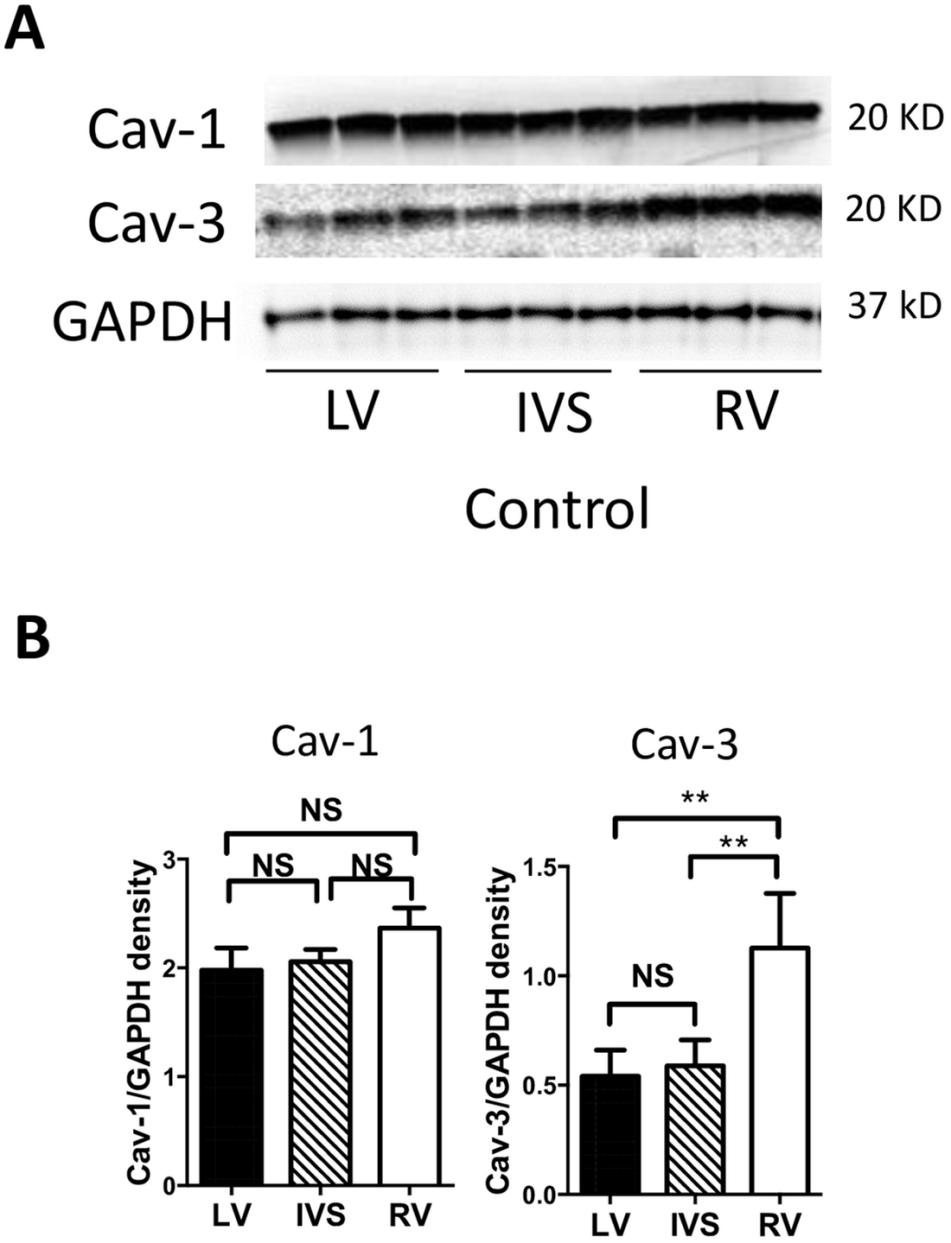
Expression of caveolin in the control heart. (A) Western Blot for caveolin-1 (Cav-1) and caveolin-3 (Cav-3) in rabbit left ventricle (LV), intraventricular septum (IVS) and right ventricle (RV). (B) Quantification of Cav-1 and Cav-3, n = 6, respectively. **p<0.01. GAPDH, Glyceraldehyde 3-phosphate dehydrogenase; NS, not significant.

Given the potential role of caveolins in the pathogenesis of heart failure we examined the expression of caveolin isoforms after Dau exposure. Caveolin-1 and -3 are expressed in the heart, however the relative distribution of caveolin isoforms in the left and right ventricle (RV) has not been assessed. We first measured Cav-1 and Cav-3 protein expression using Western blotting of LV, intraventricular septum (IVS) and RV in control rabbits (Fig 2A and 2B). Expression of Cav-3 was higher in the RV than in the LV or IVS. However, no difference in Cav-1 protein expression was observed among the three regions.

**Fig 3:**
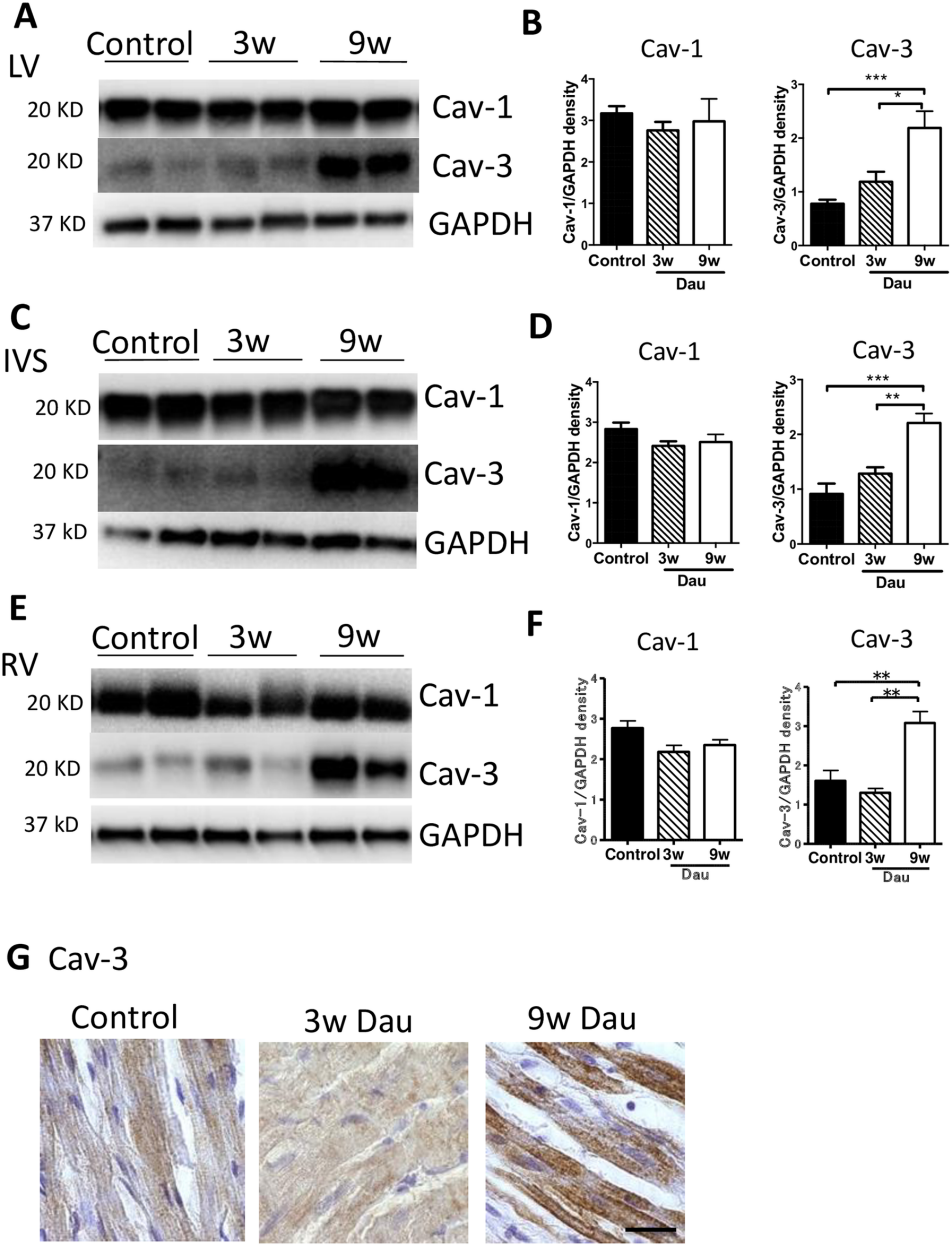
Expression of caveolin in the Dau-treated heart. (A) Western blot of Cav-1 and Cav-3 in control and Dau-treated left ventricle (LV). (B) Quantification of Cav-1 and Cav-3 in the LV, n = 6, respectively. (C) Western blot of Cav-1 and Cav-3 in control and Dau-treated intraventricular septum (IVS). (D) Quantification of Cav-1 and Cav-3 in the IVS, n = 6, respectively. (E) Western blot of Cav-1 and Cav-3 in control and Dau-treated right ventricle (RV). (F) Quantification of Cav-1 and Cav-3 in the RV, n = 6, respectively. (G) Representative images of immunostaining for Cav-3 in the control rabbit LV and Dau-treated rabbit hearts. Scale bar; 20 μm *p<0.05, **p<0.01, ***p<0.001. GAPDH, Glyceraldehyde 3-phosphate dehydrogenase; NS, not significant.

Next, we examined whether Dau treatment affected the expression of Cav-1 and Cav-3 in the heart (Fig 3A–3F). Hearts sampled after 9 weeks of Dau treatment showed increased Cav-3 expression in the LV, IVS and RV, compared to control tissues, whereas Cav-3 in 3w Dau group was unchanged. In contrast to Cav-3, Cav-1 was not changed in any experimental group.

We next examined the immunomicroscopic localization of Cav-3 protein within the heart. Hearts from 9w Dau-treated rabbits had a strong immunoreaction for Cav-3 (Fig 3G). As predicted from the fact that Cav3 is expressed primarily in muscle cells, Cav-3 was localized in the cardiac myocytes. These results suggest that when heart failure is induced by 9 weeks of Dau treatment, Cav-3 expression is increased in the rabbit heart.

**Fig 4:**
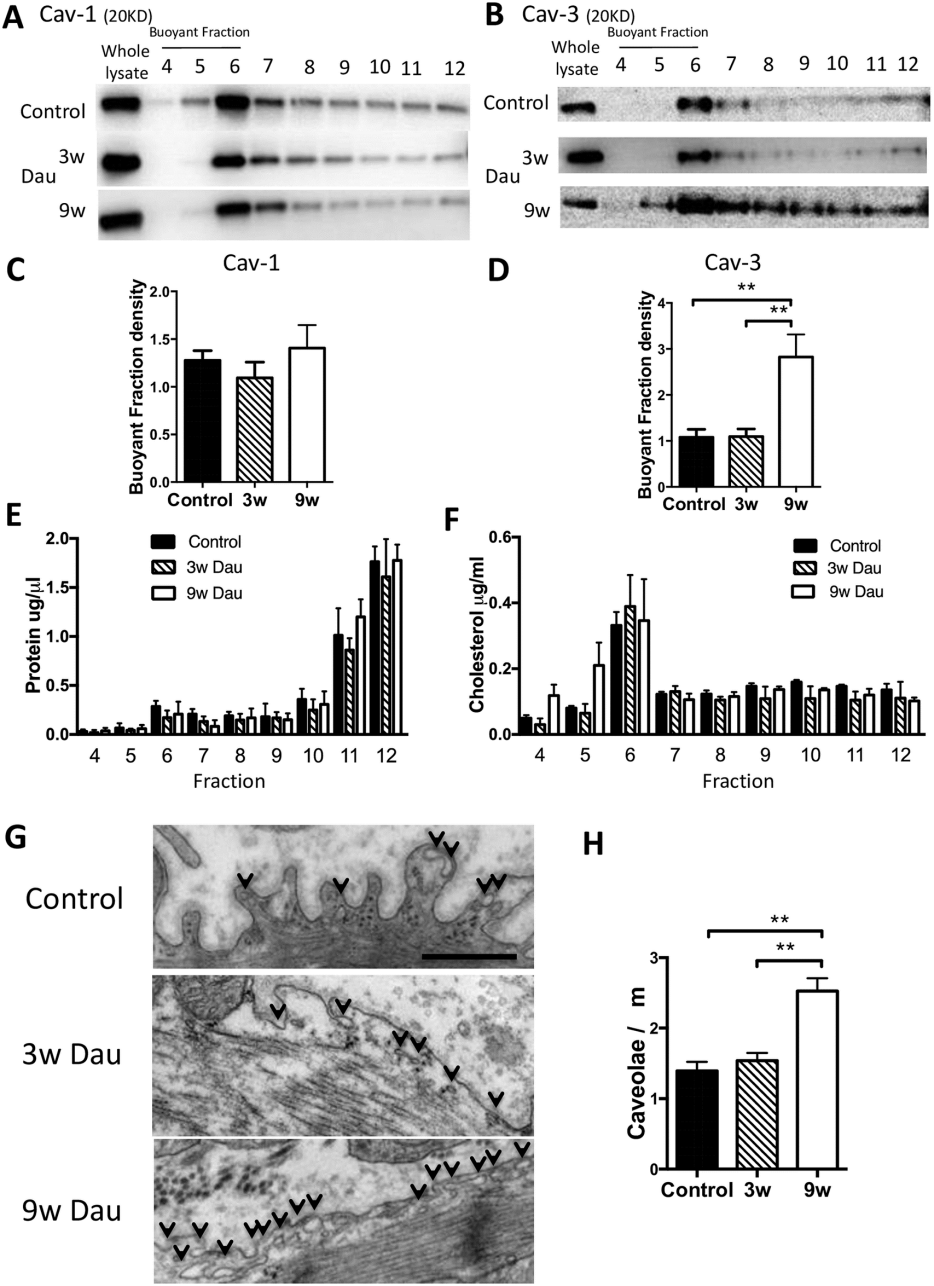
Caveolae number in cardiac myocyte from Dau-treated and control hearts. (A-B) Excised control and Dau-treated hearts underwent sucrose density fractionation. Fractions were probed for Caveolin-1 (Cav-1) and Caveolin-3 (Cav-3). Cav-3, but not Cav-1, was increased in BFs in 9w Dau-treated heart (representative immunoblots are shown) and confirmed by densitometry normalized to whole tissue lysate. (C-D) Quantification of (A) and (B). n = 4, respectively (E-F) Protein and cholesterol concentration in each fraction. n = 4, (G) 9w Dau treatment increased the number of caveolae. Electron microscopy showed an increase in number of caveolae in 9w Dau-treated vs. control hearts. Arrow indicates caveolae. Scale bar; 500 nm, (H) Caveolae number in Dau-treated and control left ventricle. (n = 10–13, respectively) *p<0.05, **p<0.01, NS, not significant; BF, buoyant fraction; non BF, non-buoyant fraction.

To expand our initial findings, hearts from Dau-treated and control rabbits were fractionated on a discontinuous sucrose gradient and analyzed for distribution of caveolin proteins. Cav-3 was unchanged after 3 weeks of Dau treatment and increased by 9 weeks of Dau treatment in buoyant fractions (BF) (numbers 4 through 6 in the 12-step gradient) (Fig 4B and 4D). No significant difference in Cav-1 protein expression was observed in buoyant membrane fractions at any time point (Fig 4A and 4C). Cholesterol content and protein concentration were not changed in any of the experimental groups (Fig 4E and 4F).

### Caveolae in the heart after daunorubicin

Since caveolin proteins are essential for formation of caveolae, we next evaluated caveolae formation by electron microscopy. Representative electron microscopy images show that formation of caveolae were easily observed in Dau-treated and control rabbits (Fig 4G). The number of caveolae was increased significantly in cardiac myocytes from the rabbits in the 9w Dau group compared with the control rabbits and rabbits in 3w Dau group (Fig 4H).

### Mitochondrial number, size and protein expression in the heart after daunorubicin

**Fig 5:**
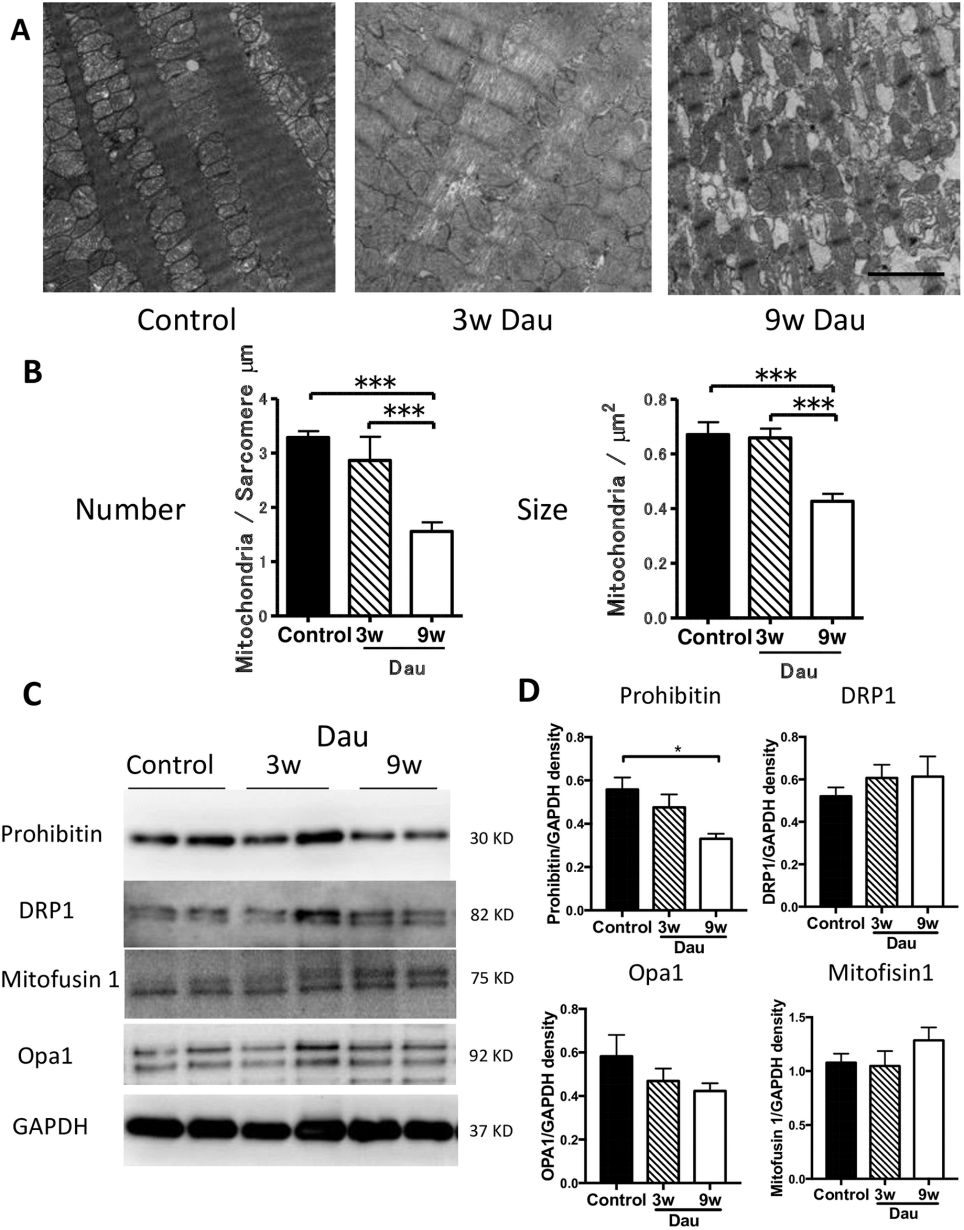
Mitochondrial changes in the Dau-treated heart. (A-B) Representative electron microscopic images of LV from Dau-treated and control hearts. Mitochondrial number and size were decreased after 9w Dau treatment when compared to mitochondria in control rabbit hearts. Scale bar = 2 μm (n = 14–17 and n = 41–53) (C) Western Blot of mitofusin-1, Opa-1, prohibitin and DRP-1 in LV from Dau-treated and control hearts. (D) Quantification of (C), n = 4–6, respectively. *p<0.05, ***p<0.001. LV, left ventricle; GAPDH, Glyceraldehyde 3-phosphate dehydrogenase; NS, not significant.

Next we determined the impact of Dau on the intracellular ultrastructure of the heart. Given that Dau increases generation of reactive oxygen species (ROS) we focused on cardiac mitochondria. In the rabbit hearts, many mitochondria were found around the sarcolemma (Fig 5A). After three weeks of Dau treatment, mitochondrial size and mitochondrial number were maintained. Both mitochondrial number and mitochondrial size were decreased after 9 weeks of Dau treatment compared to control hearts (Fig 5B). We assessed changes in mitochondrial fusion/fission and turnover by measuring the expression of fission (Drp1) and fusion (mitofusin1 and Opa1), prohibitin proteins. Expression of prohibitin in the LV after 9 weeks of Dau was lower than that of control hearts. No difference in mitofusin1, Drp-1 and Opa1 was observed in the LV among the experimental groups (Fig 5C and 5D).

### Integrin expression in the heart after daunorubicin

**Fig 6:**
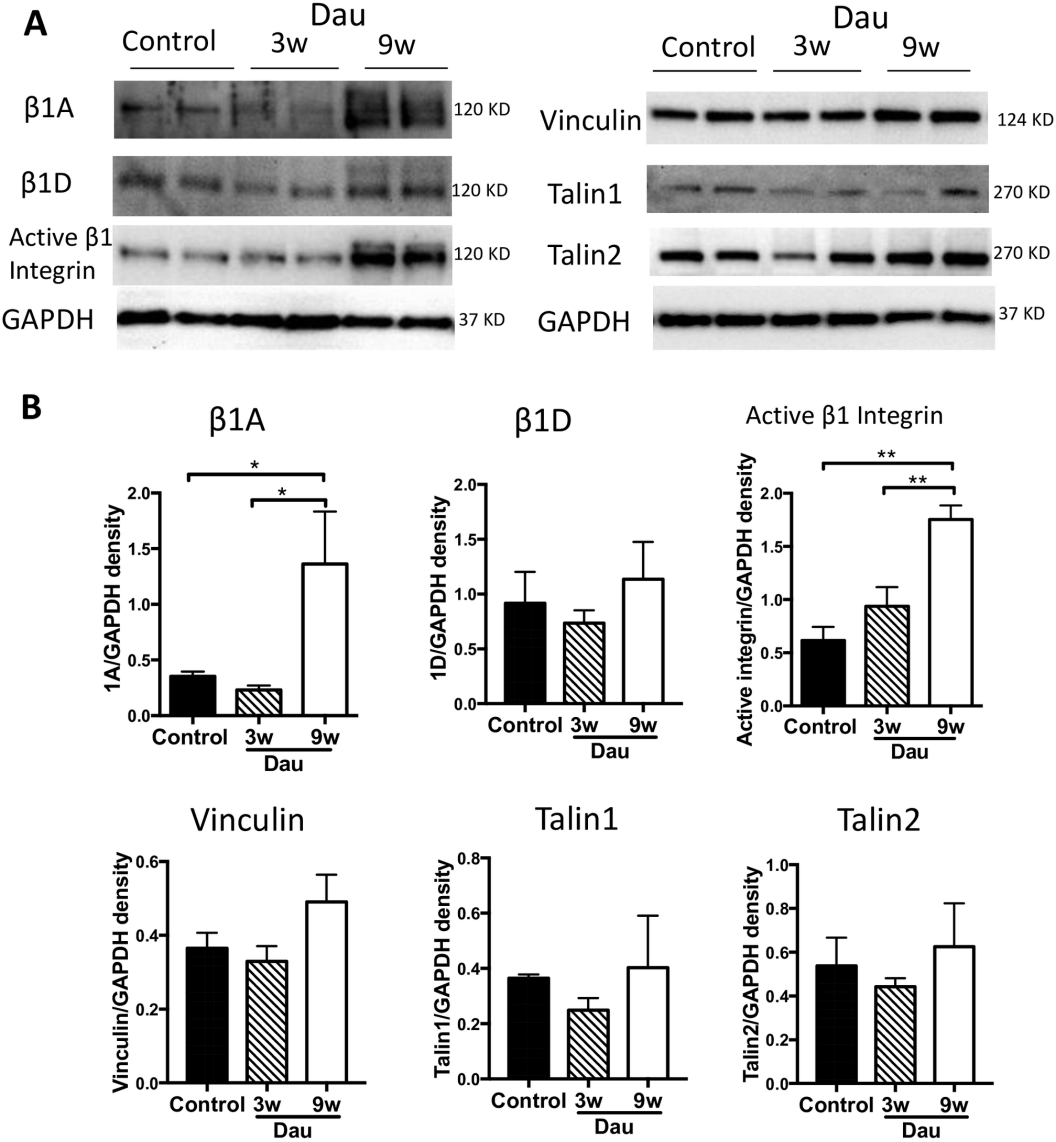
Integrins and related proteins in the Dau-treated heart. (A) Western blot of active β1 integrin, β1D, β1A, talin-1, talin-2, and vinculin in LV from Dau-treated and control hearts. (B) Quantification of (A), n = 4–6, respectively. *p <0.05, **p<0.01. GAPDH, Glyceraldehyde 3-phosphate dehydrogenase.

In a previous study we showed that Cav-3 was involved in integrin function and activation in cardiac myocytes [18]. We therefore investigated LV integrin expression and activation after Dau. First, we measured active and total β1 integrin, using specific antibodies for each form (β1A, β1D, active β1 integrin), as β1 is the dominant β subunit in cardiac myocytes (Fig 6A and 6B). Protein expression of active β1 integrin in LV was increased after 9 weeks of Dau treatment. Total β1A integrin (the ubiquitous β1 isoform) also was increased which reflects cardiac remodeling. No changes in total β1D integrin (the muscle dominant β1 isoform) expression were observed after 9 weeks of Dau when compared to control hearts and 3 weeks of Dau treatment. We also evaluated integrin-related proteins (Fig 6A and 6B), including talin and vinculin. There were no significant changes in talin-1, talin-2 or vinculin expression among the experimental groups.

### Membrane repair proteins in the heart after daunorubicin

**Fig 7:**
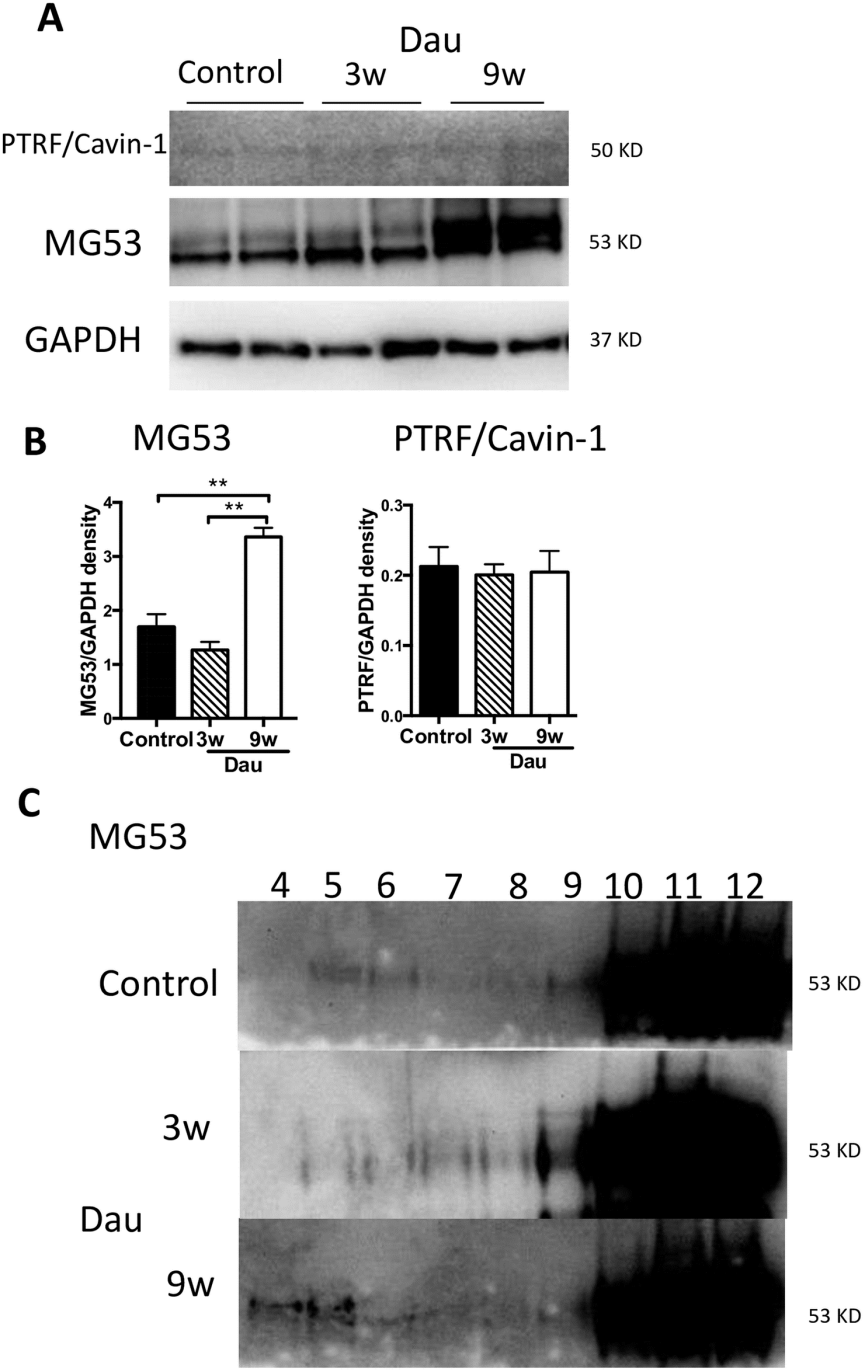
Membrane repair proteins in the Dau-treated heart. (A) Western blot of MG53, PTRF/cavin-1 in rabbit left ventricle (LV) from Dau-treated and control heart. (B) Quantification of (A), n = 4–6, respectively. Expression of MG53 in the 9w Dau-treated LV was higher than that of control LV. n = 6, respectively (C) Whole heart protein homogenates were biochemically fractionated by sucrose gradient fractionation. MG53 was found in buoyant as well as non-buoyant fractions in LV from Dau-treated and control rabbits. *p<0.01, NS, not significant; MG53, Mitsugumin-53; GAPDH, Glyceraldehyde 3-phosphate dehydrogenase.

Given that Dau may provoke membrane damage, we next evaluated membrane repair associated proteins. Cav-3 has been shown to be an interacting partner of MG53, which has been implicated in membrane repair processes. We measured protein expression of MG53 in Dau-treated hearts. After 3 weeks of Dau treatment MG53 was unchanged. After 9 weeks of Dau treatment, MG53 in the LV was increased (Fig 7A and 7B). PTRF/cavin-1 was not significantly different at either Dau-treated time point or between Dau-treated or control rabbits. We confirmed that MG53 was present in buoyant membrane fractions where Cav-3 is present (see Fig 4B) in hearts at both Dau-treated time points and in control hearts. However, the majority of the MG53 protein was located in heavy membrane fractions (Fig 7C).

## Discussion

Heart failure produced by chemotherapeutics is an increasingly important clinical concern [2]. Extensive study of anthracycline-induced cardiotoxicity has been conducted predominantly in small rodents. We developed a reproducible model of heart failure induced by daunorubicin (Dau) administration in rabbits, to increase the clinical relevance and therapeutic potential of the model. We sought to examine how caveolins, integrins and membrane repair proteins would be affected by anthracyclines given the important interactions of these proteins in the pathophysiology of heart failure [18,27]. To our knowledge only one previous publication has examined a role for Cav-1 and Cav-3 in anthracycline cardiotoxicity. This group showed that Cav-1 and Cav-3 are required for doxorubicin-induced apoptosis in atrial cardiomyocytes [28]. The expression and interaction of caveolins, integrins and membrane repair proteins has not been addressed in anthracycline-induced heart failure. We show that heart failure with reduced ejection fraction can be induced in rabbits with nine weeks of exposure to Dau. Additionally, we show that expression of Cav-3, activated β1 integrins and membrane repair proteins are increased in hearts after nine weeks of Dau treatment.

Anthracyclines are an important component of many chemotherapy protocols but utility is limited by dose-dependent cardiotoxicity [3]. Doxorubicin and daunorubicin have similar anticancer effects, though Dau reaches higher peak intracellular concentrations than doxorubicin in leukemic cells [29]. Daunorubicin is less cardiotoxic than doxorubicin among survivors of human pediatric cancer [30–32]. In rabbits, Dau produces less nephrotoxicity compared to doxorubicin [33]. Many previous studies of anthracycline cardiotoxicity used doxorubicin in rodents, primarily administered via intraperitoneal injection [7]. We chose to evaluate Dau via intravenous administration in rabbits given its favorable cardiotoxic equivalence and reduced off target side effects when compared to doxorubicin. Further understanding of the pathophysiology of anthracycline-induced cardiotoxicity has potential to produce new therapeutic options.

Currently, treatments for anthracycline-induced cardiotoxicity follow standard therapies for congestive heart failure [34]. Having more detailed information about the mechanisms that cause this toxicity could lead to targeted therapies. Caveolins play a major role in cardiac physiology and pathophysiology [10,23,25,35,36], yet the role of caveolins in heart failure is an area of continued research and debate. Studies have shown a wide variation in the expression levels of caveolins and specifically Cav-3 in different heart failure models, with studies showing Cav-3 expression being increased [37–39], decreased [11,40,41] or unchanged [42]. The reasons for the differences in caveolin expression are not known, though variations in the species studied, model of cardiomyopathy, and the degree and chronicity of the heart failure may play a role. Importantly, in the present study we show for the first time that structural caveolae and Cav-3 protein are both increased after nine weeks of Dau treatment in rabbits. In contrast to our findings, other investigators have shown that in rabbit models of pressure overload heart failure Cav-3 expression is decreased [43]. Interestingly, Cav-1 was not changed in the Dau rabbit model we employed, despite previous findings that anthracyclines can up-regulate Cav-1 expression in cancer cells [16,17].

We also showed changes in mitochondrial size and number in the Dau-treated rabbit heart. In highly metabolic organs like heart, mitochondria play a critical role in the adaptation to cellular injury [44]. Changes in mitochondrial morphology may result in increased generation of ROS and decreased respiratory function [15]. It is known that anthracycline-induced cardiotoxicity involves the generation of excess ROS by electron exchange between the anthracycline quinone moiety, oxygen molecules and other cellular electron donors [45]. ROS induces multiple forms of cellular damage to cardiac myocytes, linked to excess free radical production [2]. Previously we showed that overexpressing Cav-3 in cardiac myocytes reduced generation of ROS [15] and protected the heart from ventricular pressure overload [10], ischemia-reperfusion injury [46], and ischemia-reperfusion induced apoptosis [47]. Since we have not measured ROS generation directly, the potential role for Cav-3 modulating ROS generation in response to daunorubicin remains speculative and will require further study. We examined two time points after exposure to Dau, three and nine weeks. At the three-week time point heart function, mitochondrial number and size were preserved. However, at the nine-week time point physiological and histological changes in the heart and mitochondria progressed. In the present study, Cav-3 was unchanged after three weeks of exposure to Dau and was increased after nine weeks of Dau treatment. The present study suggests that Cav-3 expression and caveolae number are increased after nine weeks of Dau treatment in the rabbit heart, but not in sufficient amounts to prevent changes in mitochondrial morphology and presumably generation of ROS and respiratory function. Future studies should evaluate if early overexpression of Cav-3 is protective against Dau-induced cardiotoxicity.

Caveolins interact with membrane proteins including integrins and membrane repair proteins. Further, caveolins can modify integrin function in cardiac myocytes [18]. We found integrins were activated in the nine week Dau treated hearts, which may indicate a protective mechanism that the myocyte uses to adhere more firmly to matrix in the face of stress. Plasma membrane repair is critical for cellular homeostasis and prevention of cell death in the heart [19]. MG53 is a muscle-specific TRIM-family protein (TRIM72) that forms a functional complex with Cav-3 in the heart and contributes to intracellular vesicle trafficking and myogenesis [27]. Polymerase-1 and transcriptase release factor (PTRF)/cavin-1 also is a key molecule in plasma membrane repair [19]. PTRF/cavin-1 is essential for multi-drug resistance responses in cancer cells [8]. MG53 participates in the cardioprotection associated with ischemic postconditioning through tethering Cav-3 and phosphoinositide 3-kinase (PI3K) and activation of the reperfusion injury salvage kinase pathway [27,48]. A recent report also showed that MG53 was an effective biomarker of myocardial injury and dysfunction in rodent models. However in that study, MG53 expression in the human heart was low suggesting that the role of MG53 as a clinical biomarker of myocardial injury will require further investigation [49]. Despite evidence for clear interactions between caveolins, integrins and membrane repair proteins there are no prior studies that have linked the effects of anthracyclines to all these membrane structural proteins. Our results imply that up-regulation of Cav-3, activated integrins, and MG53 in our Dau-induced cardiotoxicity model may reflect the critical need for membrane repair processes when anthracycline-induced membrane injury occurs. Future work is warranted to delineate the role of these associated proteins and membrane repair in general in anthracycline-induced cardiotoxicity.

In summary, we have described the cardiac consequences of short and longer- term anthracycline exposure and ultimately anthracycline-induced cardiotoxicity in rabbits. Our results suggest a new mechanistic paradigm for anthracycline-induced cardiotoxicity involving membrane-associated proteins such as caveolins, integrins and proteins involved in membrane repair. These data add to the understanding of the pathophysiology of anthracycline-induced cardiotoxicity and could lead to the development of novel future therapies.

## Supporting information

S1 File Original gel and band. (DOCX)

S2 File Raw data. (XLSX)
