# Supplementary figures and images for: Modulation of caveolins, integrins and plasma membrane repair proteins in anthracycline-induced heart failure in rabbits
Source: PLoS One. 2017 May 12;12(5):e0177660. doi: 10.1371/journal.pone.0177660 (PMC5428970; doi:10.1371/journal.pone.0177660)

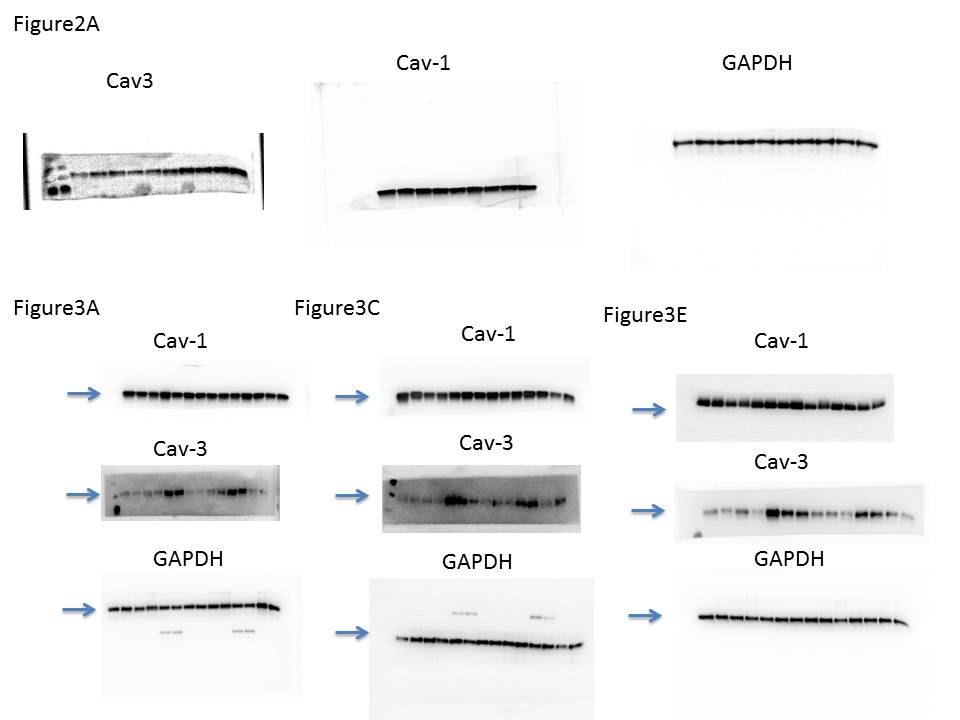


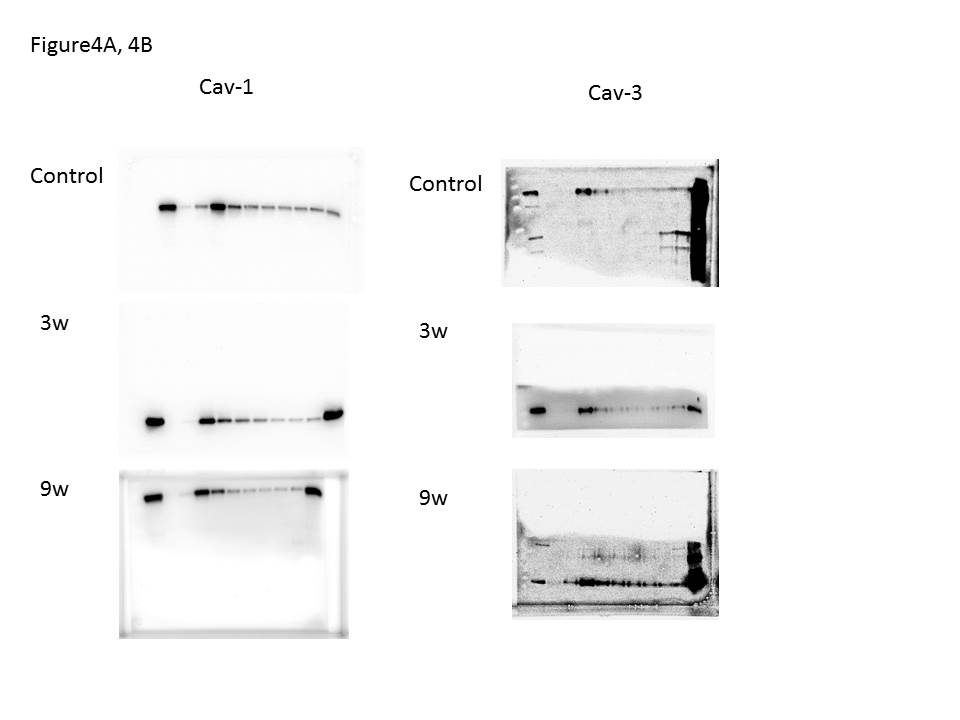


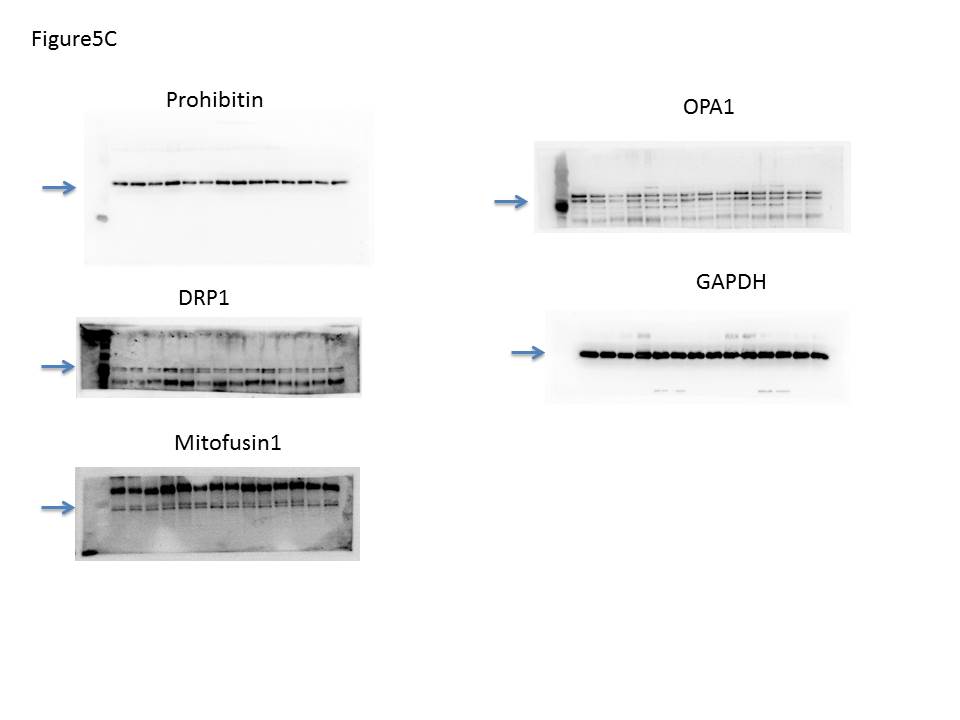


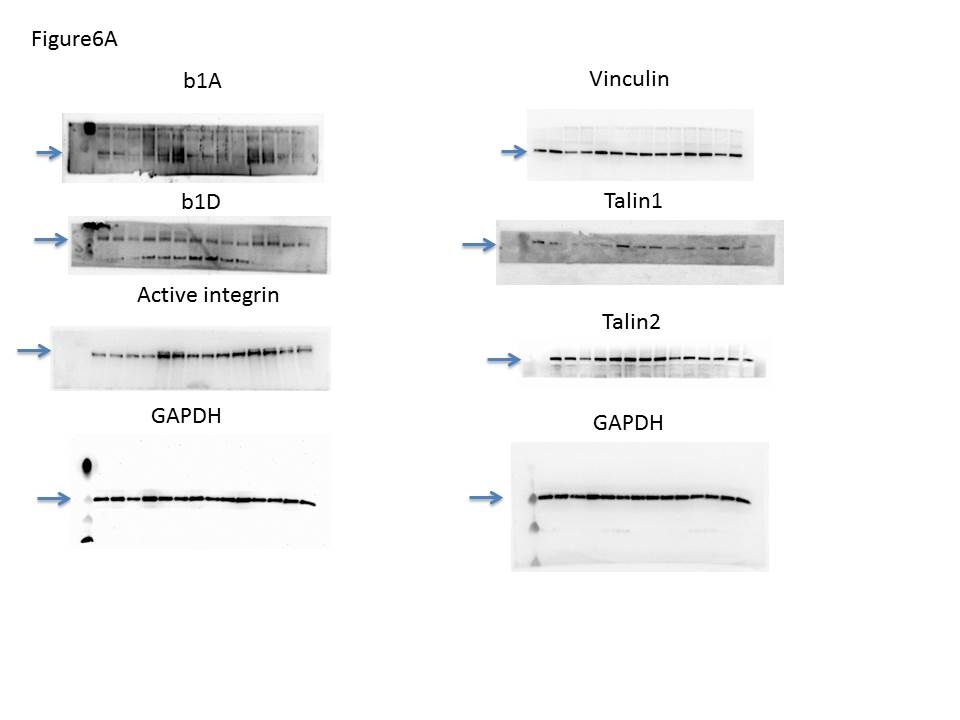


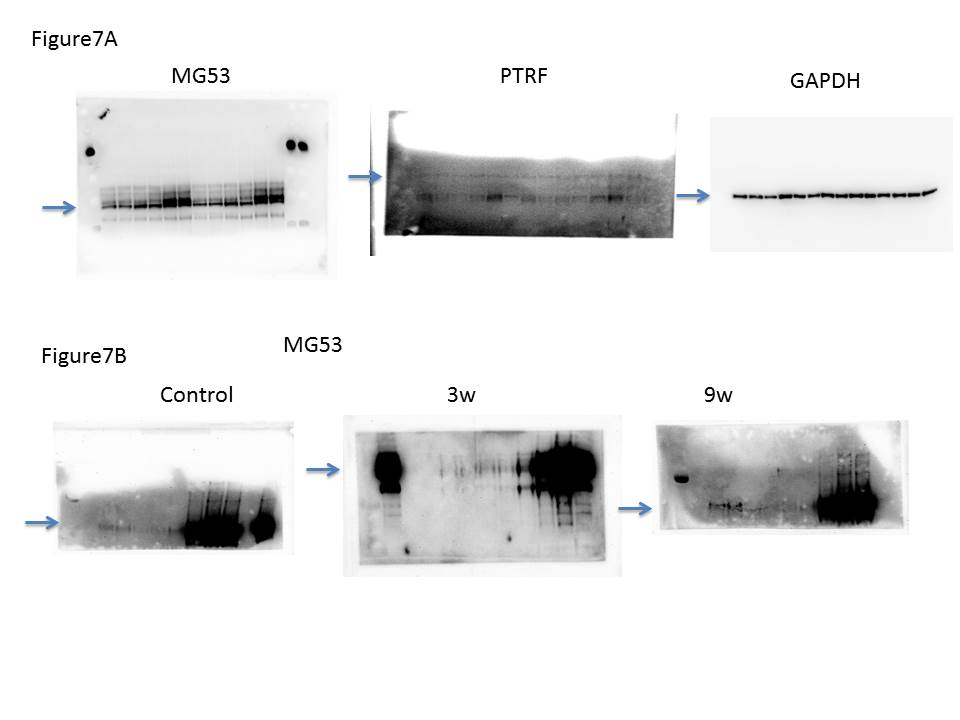

Supplement: S1 File — (DOCX) [file pone.0177660.s001.docx]
